# Assessing the Validity of Using Serious Game Technology to Analyze Physician Decision Making

**DOI:** 10.1371/journal.pone.0105445

**Published:** 2014-08-25

**Authors:** Deepika Mohan, Derek C. Angus, Daniel Ricketts, Coreen Farris, Baruch Fischhoff, Matthew R. Rosengart, Donald M. Yealy, Amber E. Barnato

**Affiliations:** 1 Department of Critical Care Medicine, University of Pittsburgh School of Medicine, Pittsburgh, PA, United States of America; 2 RAND Corporation, Pittsburgh, PA, United States of America; 3 Department of Social and Decision Sciences, Carnegie Mellon University, Pittsburgh, PA, United States of America; 4 Department of Surgery, University of Pittsburgh School of Medicine, Pittsburgh, PA, United States of America; 5 Department of Emergency Medicine, University of Pittsburgh School of Medicine, Pittsburgh, PA, United States of America; 6 Department of Medicine, University of Pittsburgh School of Medicine, Pittsburgh, PA, United States of America; University of Washington, United States of America

## Abstract

**Background:**

Physician non-compliance with clinical practice guidelines remains a critical barrier to high quality care. Serious games (using gaming technology for serious purposes) have emerged as a method of studying physician decision making. However, little is known about their validity.

**Methods:**

We created a serious game and evaluated its construct validity. We used the decision context of trauma triage in the Emergency Department of non-trauma centers, given widely accepted guidelines that recommend the transfer of severely injured patients to trauma centers. We designed cases with the premise that the representativeness heuristic influences triage (i.e. physicians make transfer decisions based on archetypes of severely injured patients rather than guidelines). We randomized a convenience sample of emergency medicine physicians to a control or cognitive load arm, and compared performance (disposition decisions, number of orders entered, time spent per case). We hypothesized that cognitive load would increase the use of heuristics, increasing the transfer of representative cases and decreasing the transfer of non-representative cases.

**Findings:**

We recruited 209 physicians, of whom 168 (79%) began and 142 (68%) completed the task. Physicians transferred 31% of severely injured patients during the game, consistent with rates of transfer for severely injured patients in practice. They entered the same average number of orders in both arms (control (C): 10.9 [SD 4.8] vs. cognitive load (CL):10.7 [SD 5.6], p = 0.74), despite spending less time per case in the control arm (C: 9.7 [SD 7.1] vs. CL: 11.7 [SD 6.7] minutes, p<0.01). Physicians were equally likely to transfer representative cases in the two arms (C: 45% vs. CL: 34%, p = 0.20), but were more likely to transfer non-representative cases in the control arm (C: 38% vs. CL: 26%, p = 0.03).

**Conclusions:**

We found that physicians made decisions consistent with actual practice, that we could manipulate cognitive load, and that load increased the use of heuristics, as predicted by cognitive theory.

## Introduction

Understanding why physicians fail to follow clinical practice guidelines has occupied researchers and policy makers for the past four decades. [1]–[4] The dual-process model of cognitive reasoning may help to explain the persistent gap between normative standards and practice patterns. [5] This model posits that two systems of cognitive operations shape judgments: system one (heuristic) processes function intuitively, relying on pattern recognition for answers; system two (analytic) processes function more laboriously, incorporating rule-based deductions. The relative weight exerted by these two systems depends on the characteristics of the task (time-pressure, cognitive load) and the person making the judgment (expertise, motivation). [6] Understanding the roles of these different processes is vital for improving decision making.

People have imperfect introspective access to their own judgment, and so cannot accurately explain why they do things. [7] As a result, researchers must draw inferences about cognitive processes by examining decisions made under systematically varying conditions. Existing methods have known limitations. For example, paper vignettes with static descriptions have unclear external validity; direct observation rarely allows manipulation of task conditions; live simulation requires costly investments in infrastructure and data collection. [8]–[12] We offer a new tool for studying those processes, serious games, which use gaming technology to create realistic versions of clinical problems. Serious games can, in principle, retain clinical validity by simulating representative task environments, while allowing the experimental manipulation of task conditions that can reveal cognitive processes [13]. After the initial investment in their creation, they can be implemented relatively inexpensively. However, little is known about their validity in this context. [14] The present study fills some of that gap.

The objective of the study was to evaluate the construct validity of a serious game for studying physician decision making, by measuring a) the game's external validity; b) our ability to manipulate one task condition, cognitive load; and c) participants' responses to those manipulation, in terms of their consistency with predictions based on cognitive theory, specifically predicting increased reliance on heuristics under greater cognitive load.

## Methods

We used the decision context of trauma triage in the Emergency Department of non-trauma centers, and recruited a convenience sample of physicians at a national meeting of the American College of Emergency Physicians in the fall of 2013.

### Conceptual Model

Trauma triage exemplifies decisions made under time pressure and uncertainty, where physicians' abilities to identify rare events have important implications for patient outcomes. Moreover, it is a context where practice patterns often deviate from guidelines, despite intensive quality improvement interventions by major professional stakeholders. [15]–[17] The American College of Surgeons provides widely-known guidelines for identifying severely injured patients who should receive care at trauma centers. [15] However, among patients with severe injuries who present initially to non-trauma centers, only one-third are transferred to a higher level of care. [16], [7]

The specific hypotheses that we pursued arose from the conjunction of clinical experience, retrospective data analyses, and decision science research. [18]–[21] We developed a conceptual model of physician triage decisions as the product of judgment (information processing and probability estimation) and choice (selection between available alternatives). [22] In other words, physicians have to judge the severity of the injury, and then choose how to manage it. Judgment arises from the interaction between system one (fast, heuristic) processes and system two (slow, analytic) processes. [5] As things speed up, people rely more heavily on heuristics (or cognitive shortcuts) that are often useful, but can lead them astray. As things slow down, they are better able to synthesize the complex, uncertain elements of more difficult decisions (assuming that they have the training to do so). [6], [23] Choice reflects variables such as physician attitudes towards the guidelines, outcome expectancy, institutional norms, regional resource constraints, and patient preferences. [17], [24]

We wanted to understand the influence of system one processes on trauma triage decision making. Our prediction for the expression of heuristic thinking was based on our clinical experience. For example, we had observed that patients with gunshot wounds were far more likely to be transferred to a trauma center than patients who had fallen, for any given injury severity score. [18], [25] That pattern is consistent with reliance on the *representativeness heuristic*, whereby the likelihood of an outcome depends on how well the case fits (or represents) the process involved. [21] Thus, physicians relying on the heuristic would interpret actual cases in terms of an archetype of what happens when people are shot or fall, then use that match as their default diagnosis. They would require stronger evidence, and time to think, to conclude otherwise. To the extent that transfer decisions reflect the use of heuristics, increased cognitive load should increase adherence with clinical practice guidelines where heuristics match the guidelines (representative cases) and physicians reach the right decision more quickly, despite less thorough examination. It should decrease adherence where heuristics do not match the guidelines (non-representative cases) and physicians lack the time to double check their thinking.

### Case Development

In previous work, we developed and validated 30 static paper-based trauma case vignettes. [18], [26] We selected 12 cases (6 with severe injuries and 6 with minor injuries) and developed them into branching vignettes. Among the cases describing patients with severe injuries, we included two *representative* cases (e.g. gunshot wound to abdomen) and four *non-representative* cases (e.g. fall with multiple rib fractures). We made the patient's age and hemodynamic stability on presentation orthogonal to the representativeness of the case, so that we could distinguish the effect of the injury complex from other variables known to influence decision making. [18]–[20] We created an additional eight non-trauma cases, four with critical illnesses and four with routine complaints to serve as clinical distractors. [Table 1]

**Table 1:** Case Descriptions.

		Control	Cognitive Load
**Trauma Cases**	**Severe**	s/p fall with epidural hematoma with seizure on presentation [NR]	s/p fall with epidural hematoma with seizure on presentation [NR]
		s/p motor vehicle collision with aortic transection [NR]	s/p motor vehicle collision with aortic transection [NR]
		s/p motor cycle collision with open book pelvic fracture with hemodynamic instability on presentation [R]	s/p motor cycle collision with open book pelvic fracture with hemodynamic instability on presentation [R]
		s/p motor cycle collision with open humerus fracture with no distal perfusion [NR]	s/p motor cycle collision with open humerus fracture with no distal perfusion [NR]
		s/p pedestrian versus car with multiple rib fractures; subdural hematoma [NR]	s/p pedestrian versus car with multiple rib fractures; subdural hematoma [NR]
		s/p gunshot wound to abdomen with grade IV liver laceration and hemodynamic instability on presentation [R]	s/p gunshot wound to abdomen with grade IV liver laceration and hemodynamic instability on presentation [R]
	**Minor**	s/p bicycle v. auto with distal radius/ulnar fracture	s/p bicycle v. auto with distal radius/ulnar fracture
		s/p motor cycle collision with concussion	s/p motor cycle collision with concussion
		s/p assault with concussion	s/p assault with concussion
		s/p motor vehicle collision with chest pain	s/p motor vehicle collision with chest pain
		s/p gunshot wound to left calf, right thumb, and right buttock	s/p gunshot wound to left calf, right thumb, and right buttock
		s/p motor vehicle collision with left mandibular fracture	s/p motor vehicle collision with left mandibular fracture
**Non-trauma cases**	**Routine**	Appendicitis	
		Abscess of arm	
		Headache	
		Non-cardiac chest pain	
	**Critically ill**		Hypertensive sub-arachnoid hemorrhage
			Decompensated congestive heart failure with respiratory failure
			Diverticular bleed with hemodynamic instability
			Sepsis with hemodynamic instability

NR – non-representative; R – representative.

### Serious game development

The term ‘serious games’ refer to all computer games that have a purpose other than pure entertainment, such as education, behavior change, or scientific research. [14] These games can range from virtual simulations to more imaginary or abstract tasks. However, they rely on the engagement and challenge of game play to facilitate their objectives. [13], [14]

**Figure 1:**
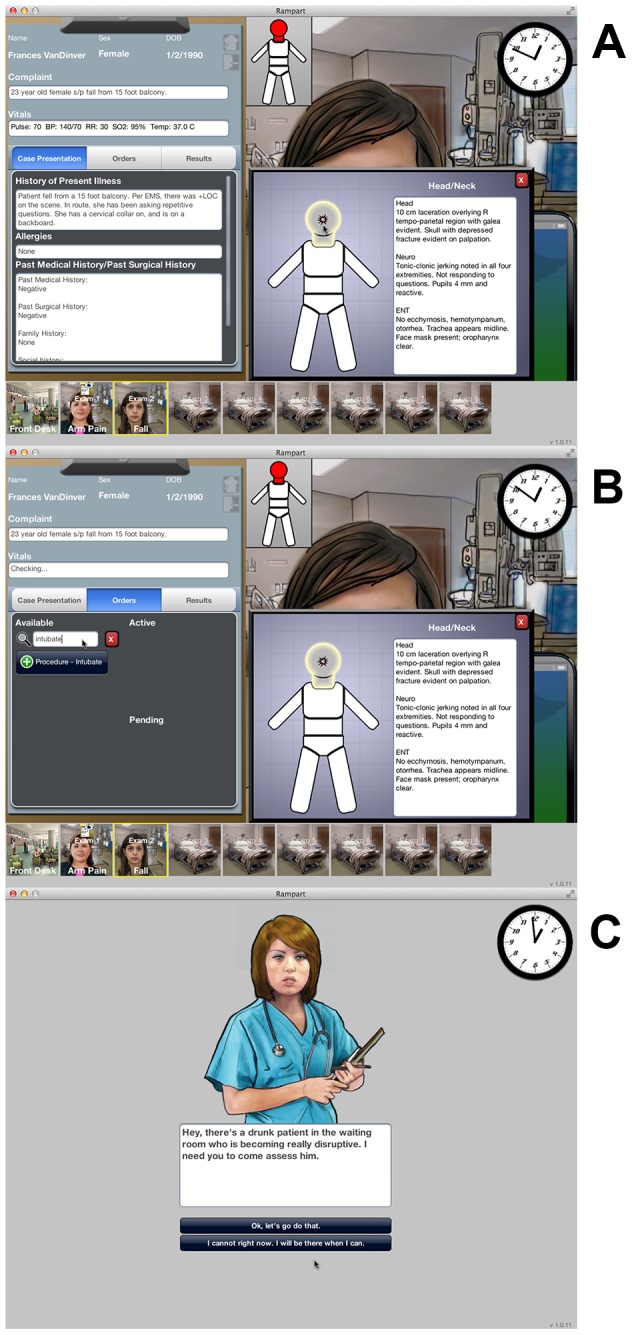
Screen shots of serious game. A) Each case included a 2-D rendering of the patient, a chief complaint, vital signs, a history, and a written description of the physical exam. Physicians had 42 minutes (a simulated 8 hour shift) to complete the ten cases. A clock at the top right of the screen helped track the passage of time. B) Physicians could manage patients by selecting from a pre-specified list of 250 medications, studies, and procedures. C) We included audio-visual distractors, including nursing requests for help with disruptive patients to increase the verisimilitude of the experience.

Our serious game simulates the environment of an Emergency Department (ED) at a non-trauma center. We collaborated with a gaming company (Breakaway Ltd; Hunt Valley, MD) to transfer the paper vignettes into a 2-D serious game. Participants had to evaluate and manage ten cases over 42 minutes, simulating a busy eight-hour ED shift. New patients arrived at pre-specified (but unpredictable) intervals, so that physicians had to manage multiple patients concurrently. Each case included a 2-D rendering of the patient, a chief complaint, vital signs which updated every 30 seconds, a history, and a written description of the physical exam.[Figure 1A] In the absence of clinical intervention by the physician “player,” severely injured patients and critically ill distractor patients decompensated and died over the course of the game.

Physicians indicated their decisions about how to manage patients by selecting from a pre-specified list of 250 medications, studies, and procedures.[Figure 1B] Some orders affected patients' clinical status, leading to corresponding changes in their vital signs and physical exam. Other orders generated additional information, presented as reports added to the patients' charts. The cases ended when physicians either made a disposition decision (admit, discharge, transfer) or the patient died.

We included several design elements to enhance the verisimilitude of the experience. For example, in addition to their clinical responsibilities, players also had to respond to audio-visual distractors, including nursing requests for help with disruptive patients, interruptions by families asking for information, and paging alerts from administrators.[Figure 1C] We returned new information in a time-delayed fashion, corresponding to that in actual practice (but scaled to simulation time). For example, the results of a chest x-ray returned after two minutes, while the results of a CT scan required a five-minute wait. A clock at the top of the screen helped players to track the passage of time. We did specify that physicians could not make a disposition decision for hemodynamically unstable patients (e.g. systolic blood pressure <90), in order to prevent physicians from deflecting responsibility for potentially challenging cases. Finally, we provided no incentives beyond physicians' intrinsic desire to perform well.

### Game pre-testing

One person (DM) developed the branching vignettes based on existing validated vignettes (trauma cases) and case histories (non-trauma cases), and edited them based on the input of six content experts (three trauma surgeons and three emergency medicine physicians). After iterative pre-tests of game elements, we beta-tested a prototype among emergency medicine physicians practicing at non-trauma centers (N = 30). In response to feedback, we added additional orders, changed the clinical parameters of two cases, reduced the number of distractors, and increased the duration of the simulation from 28 to 42 minutes.

### Demographic survey

Each physician completed a 16-item questionnaire assessing age, sex, race, educational background (board certification, ATLS certification, years since completion of residency), and practice environment (trauma designation of their hospital, affiliation with a Level I/II trauma center, affiliation with an EM residency program, presence of consultants [e.g. orthopedic surgeons]).

### Experimental protocol

After subjects logged in, they completed the demographic questionnaire and then were randomized to the control arm or to the experimental arm of cognitive load. We operationalized cognitive load in two ways. First, we varied the complexity of the non-trauma cases. In the control arm, non-trauma cases had routine complaints (e.g. appendicitis), arrived hemodynamically stable, and did not deteriorate over the course of the game. In the cognitive load arm, non-trauma cases were critically ill (e.g. had sepsis), arrived hemodynamically unstable, and deteriorated without adequate management. Second, we reduced the number of rooms that physicians could use to evaluate patients, from eight in the control arm to four in the cognitive load arm. As a result, physicians in the cognitive load arm received an increased number of reminders from the nurse to complete cases because of patients queuing in the waiting room.

Physicians evaluated ten cases: three severely injured patients (one representative, two-non-representative), three minimally injured patients, and four non-trauma cases (either all with critical illness or all with routine complaints).[Table 1] After physicians completed all ten cases, or the eight-hour shift ended, they had the opportunity to provide feedback about the game. Physicians who completed the study received a $100 honorarium.

### Statistical Analyses

#### Physician Sample

We calculated the response rate as the proportion of enrolled physicians who logged into the website, and the completion rate as the proportion who completed the game. We summarized physician characteristics using means (standard deviation [SD]) for continuous variables and proportions (%) for categorical variables, and compared the distribution of characteristics between the control and experimental groups using Students t-test and chi-squares as appropriate.

#### User Experience

We categorized qualitative feedback related to the quality of the game, as positive or negative, and further categorized negative feedback as concerns about the verisimilitude of the task or technical problems when playing the game. We include a sample of participants' feedback in an Appendix.[Appendix S1]

#### Reliability

We measured the consistency with which physicians made disposition decisions for severely injured patients (internal reliability) using Cronbach's alpha coefficient.

#### Evaluating the external validity of game play

We summarized the types of diagnostic (CT scan, x-rays, labs), therapeutic (medications, procedures, consults), and disposition decisions for trauma cases. We specifically used two decisions, well-described in the literature, to evaluate the external validity of game play: the mean number of severely injured patients transferred to a trauma center and the mean number of CT scans acquired prior to transfer. [16], [17], [25]

#### Evaluating the effectiveness of the experimental manipulation

We excluded cases that were not completed at the conclusion of the game (i.e., the patient was alive but had no disposition status). We compared the number of orders, time spent on each trauma case, and types of disposition decisions in the control and cognitive load arms using Students t-test and chi-square as appropriate. We hypothesized that cognitive load would worsen performance. Given the association between delay in triage and outcome, we defined better performance as a) fewer orders entered, b) less time spent on each case, and c) more severely injured patients transferred to trauma centers as recommended by the clinical practice guidelines. We also examined the effect of cognitive load on two classes of cases for which physicians typically make the right decision easily: hemodynamic unstable ones and younger ones (<65). [19], [25]. We expected higher error rates under cognitive load.

#### Evaluating the influence of heuristics on decision making

To evaluate the influence of heuristics on decision making, we compared the mean number of transfer decisions for representative and non-representative severely injured patients using chi-square tests.

### Human Subjects and Power Calculation

We designed the experiment to capture a moderate effect (one-half a standard deviation) of cognitive load on transfer decisions for representative and non-representative cases with an alpha of 0.05 and a power of 80%. [27] Anticipating a 60% response rate, we recruited 200 physicians at a national meeting of the American College of Emergency Physicians (ACEP) in the fall of 2013. Physicians were eligible for participation if they cared for adult patients in the Emergency Department of either a non-trauma center or a Level III/IV trauma center in the United States. The University of Pittsburgh Institutional Review Board study reviewed and approved the study (IRB# PRO13090138). We conducted all analyses and data management with STATA 12/SE (College Station, TX).

## Results

### Sample

Among the 209 physicians recruited at ACEP, 168 logged on to the website (79%), and 142 (68%) completed the game. The mean age of the physicians completing the game was 43 years (SD = 10.7). 135 (95%) were Emergency Medicine residency trained, and 113 (80%) were certified in Advanced Trauma Life Support. 80 (56%) worked in a hospital affiliated with a Level I/II trauma center, 136 (96%) had a general surgeon available on call, and 72 (51%) had a neurosurgeon available. Participant characteristics did not differ between the control and cognitive load arms.[Table 2]

**Table 2:** Participant characteristics by exposure to cognitive load.

	Control n = 70	Cognitive Load n = 72	p
Demographic Characteristics			
Age (year, SD)	42 (10.2)	43 (11)	0.9
Female (n,%)	13 (19)	17 (24)	0.5
Race, (n, %)			0.32
White	52 (75)	59 (82)	
Asian	9 (13)	9 (13)	
American Indian	2 (3)	0 (0)	
Black	1 (1)	2 (3)	
Other	6 (9)	2 (3)	
Educational Training			
Completed or completing an emergency medicine residency (n, %)	68 (97)	66 (92)	0.16
Completing residency (n, %)	5 (7)	6 (8)	0.41
ATLS* certification present (n, %)	55 (79)	58 (81)	0.77
Also working at a Level I/II trauma center (n, %)	16 (23)	12 (16)	0.49
Characteristics of Practice Environment			
Is there a trauma center affiliated with their hospital (n,%)	44 (63)	36 (50)	0.12
Number of ED^°^ beds (n, SD)	35 (37)	29 (23)	0.27
Number of ICU^¶^ beds (n, SD)	22 (21)	18 (15)	0.21
Is there an EM^§^ residency at their hospital (n,%)	17 (25)	20 (28)	0.64
Do they have a general surgeon on call (n,%)	66 (95)	70 (97)	0.38
Do they have a neurosurgeon on call (n,%)	38 (54)	34 (47)	0.40
Do they have an orthopedic surgeon on call (n,%)	63 (90)	68 (94)	0.32

*ATLS = Advanced Trauma Life Support; ^°^ED = Emergency Department; ^¶^ICU = Intensive Care Unit; ^§^EM = Emergency Medicine

### User experience

**Figure 2:**
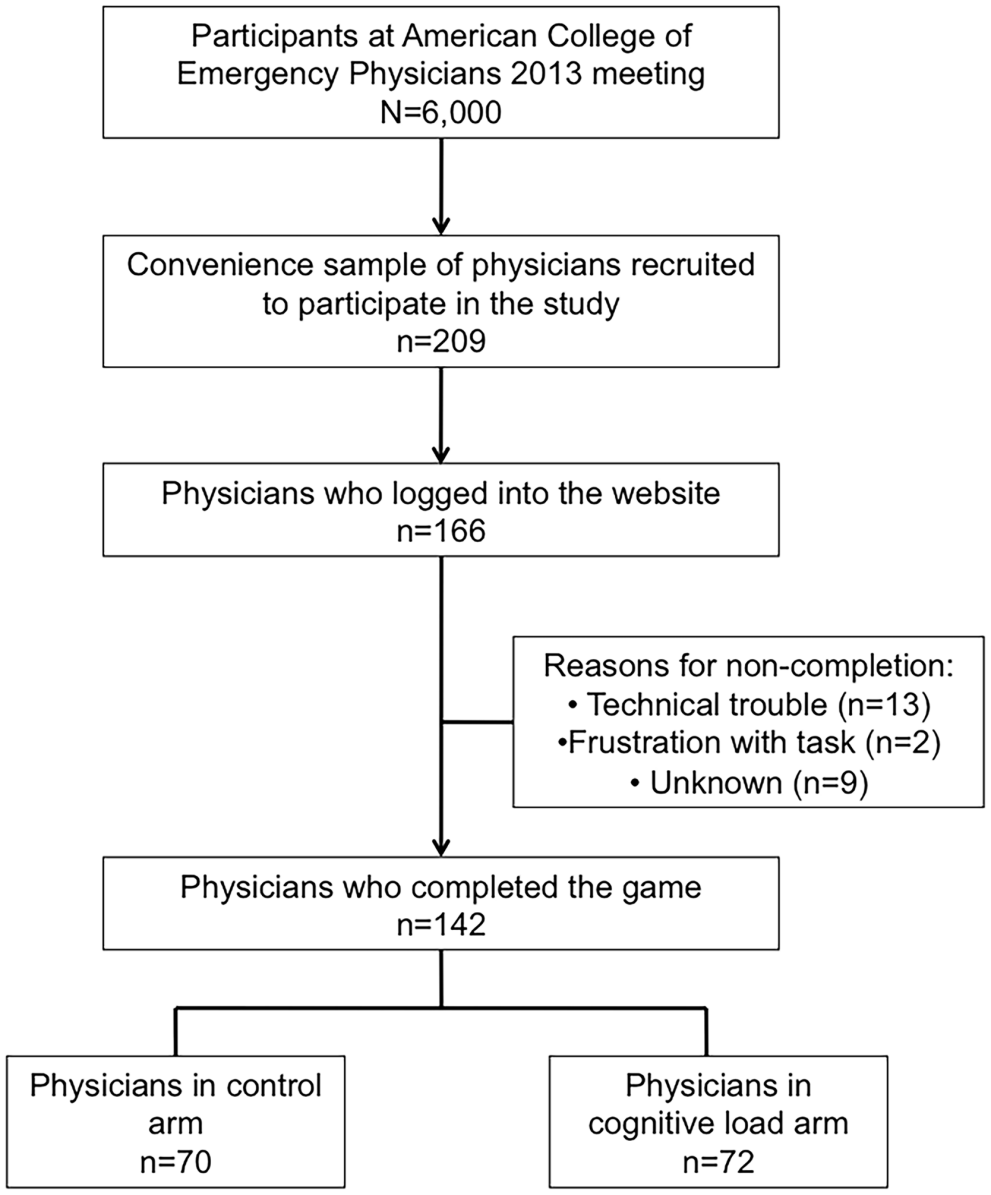
Sampling frame for study. There was a 79% response rate and an 86% completion rate.

Among physicians who completed the game, 70 (49%) were randomized to the control arm, and 72 (51%) were randomized to the cognitive load arm. [Figure 2] 71 (50%) physicians left feedback about their experience; 45 (63%) described the experience as engaging; 16 (23%) complained about verisimilitude problems with the game as a whole; and 10 (14%) described technical problems with its operation.

### Construct Validation

#### Reliability

Physicians made similar disposition decisions for cases that described severely injured patients, suggesting the instrument had acceptable internal reliability (Cronbach's alpha = 0.72).

#### Evaluating the external validity of game play

Physicians transferred 31% of severely injured patients. They obtained CT scans for 62% of patients they transferred. These results are similar to actual practice rates of 30% and 57–67%, respectively. [16], [17], [25], [29]

#### Evaluating the effectiveness of the experimental manipulation

Physicians completed 80% of the cases in the control arm and 78% in the cognitive load arm (p = 0.5). The frequency of death did not differ between arms (control (C): 7% vs. cognitive load (CL): 10%; p = 0.1). Physicians entered the same mean number of orders in both the control and cognitive load arms (C:10.9 [SD 4.8] vs. CL: 10.7 [SD 5.6], p = 0.74). However, they were more likely to make the appropriate decision to transfer severely injured patients to trauma centers in the control arm than in the cognitive load arm (C: 40% v. CL: 28%, p = 0.01). Those improved decisions occurred despite participants spending less time per case in the control arm than in the cognitive load arm (C: 9.7 [SD 7.1] vs. CL: 11.7 [SD 6.7] minutes, p<0.01).

Physicians in the control arm transferred hemodynamically unstable patients (C: 49% vs. CL: 28%, p<0.01) and younger patients to trauma centers (C: 44% vs. CL: 27%, p<0.01) more frequently than in the cognitive load arm.

#### Evaluating the influence of heuristics on decision making

**Figure 3:**
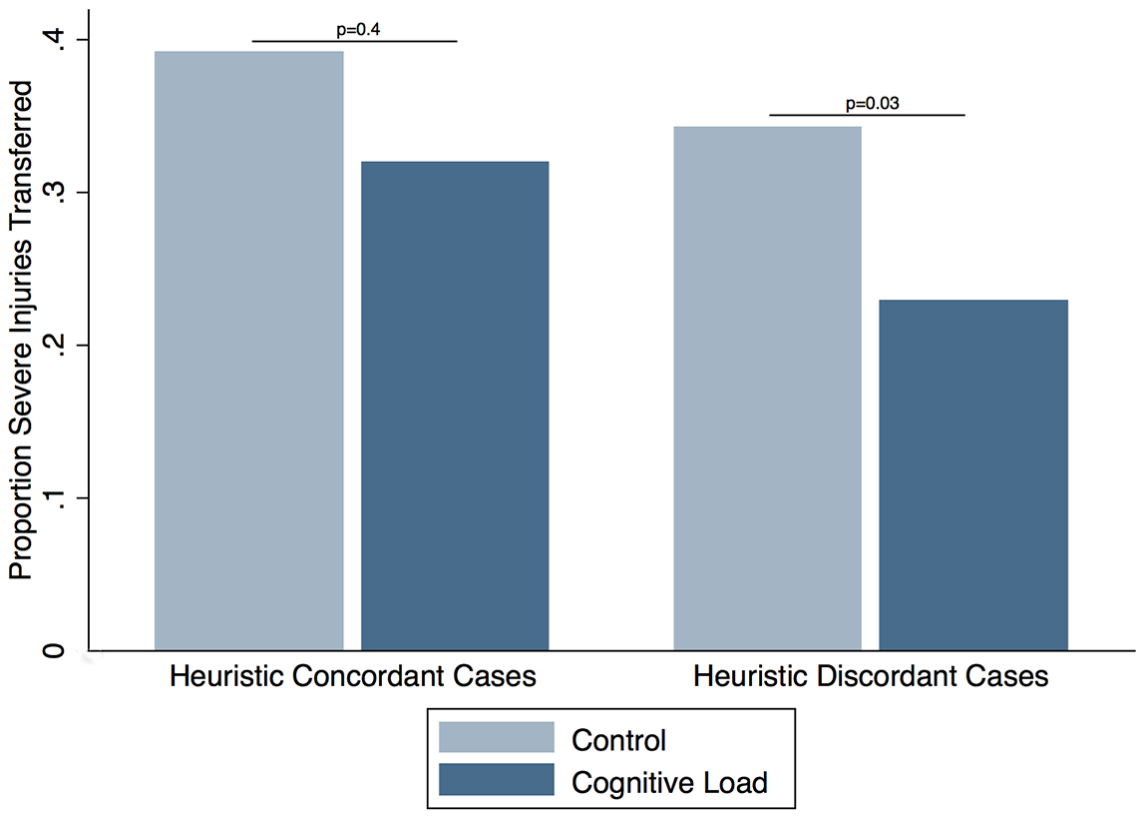
Comparison of transfer decisions for severely injured patients by physicians in the control and cognitive load arm. Physicians in the cognitive load arm were equally likely to transfer representative cases, but less likely to transfer non-representative cases.

Physicians were equally likely to transfer representative cases in the two arms (C: 45% vs. CL: 34%; p = 0.20), but were more likely to transfer non-representative cases in the control arm (C: 38% vs. CL: 26%; p = 0.03). [Figure 3]

## Discussion

In this convenience sample of ACEP attendees, key findings support the validity of using serious game technology to study physician decision making. The pattern of decisions made during the game appears consistent with actual practice patterns. The experimental manipulation of task conditions had the desired effect on cognitive load. The experimental manipulation changed decision making in ways consistent with predictions based on cognitive theory.

Existing conceptual models of physician decision making have ignored the influence of heuristics – in large part because of the lack of empirical evidence. [1] However, heuristics may play an important role in decision making, and do not respond to the same types of quality improvement interventions as other determinants. [28] Better tools for evaluating physician decision making are essential for improving the quality of performance. Here we provide proof-of-concept that serious games have the potential to serve as an important method of studying physician decision making. First, they elicit decisions consistent with actual practice. In the decision context of the ED of a non-trauma center, we found that physicians would transfer patients and obtain CT scans at the same rate as they would in real-life. [16], [17], [25], [29]

Second, serious games offer the opportunity to examine decisions under systematically varying conditions. For example, we found that we could successfully manipulate cognitive load. Limits exist for human attentional and cognitive abilities. [30] Numerous studies have demonstrated that as overall workload increases, people develop more disorganized task routines, with impaired capacity to deal with new demands. [31]–[34] We found several indicators that performance deteriorated in the experimental arm: time spent per case increased; transfer of severely injured patients (i.e. compliance with clinical practice guidelines) decreased. Additionally, during our exploration of determinants of transfer patterns, we found that errors occurred specifically for the ‘easy’ cases. In practice, physicians typically transfer younger and hemodynamically unstable patients to trauma centers. In the experimental arm of the study, the rate of transfer for these patients dropped. At first, this observation might appear paradoxical. However, the relationship between cognitive load and inattentional blindness (a failure to observe salient cues when distracted by competing demands on attention) is well-described. [35], [36] We hypothesize that treatment of young and unstable patients in the experimental arm reflects the presence of inattentional blindness, which in turn reinforces our conclusion that they experienced greater cognitive load.

Finally, by allowing the observation of decision making under different task conditions, serious games permit insight into the cognitive processes that inform judgment. We found that transfer rates of patients with severe injuries differed between arms in the study, but only for those with non-representative patterns of injuries. Based on our conceptual model of physician decision making, a number of possible explanations exist for failures to adhere to clinical practice guidelines. Physicians may choose not to follow the guidelines, either because they do not agree with them or because some external constraint (e.g. patient preferences, institutional norms) precludes adherence. If true, then cognitive load should have no influence on decision making. Alternatively, physicians may choose to follow the guidelines but find the task more difficult under conditions of cognitive load. If true, transfer patterns should vary between arms of the study: either improving under cognitive load as physicians consciously decide to respond to the challenge by sending more patients to referral centers or by worsening as physicians develop more disorganized work routines. Regardless, differences in performance should occur consistently for all cases. A third possibility is that physicians rely on their heuristics to triage patients, which do not correspond exactly with the clinical practice guidelines. If true, transfer patterns should vary between arms of the study, but only for specific types of cases (i.e. physicians will fail to transfer cases where discordance exists between their intuitive judgments about the severity of the injury and recommendations for best practice). Our observation is therefore most consistent with the increased use of the representativeness heuristic to triage patients under conditions of stress.

Our study has several limitations. Given the small number of cases, we are unable to comment on individual physicians' cognitive process, but relied on aggregate evidence. Additionally, we included a much higher proportion of severely injured patients than occurs in practice, so as to have a task of tolerable length with enough severely injured patients to evaluate our hypotheses. The effect of base rates on decision making is hard to predict. [37], [38] However, the similarity of triage rates to actual practice patterns suggests that the inflated base rate in our study did not significantly modify decision making regarding individual cases. We also have limited information about the game play experience of participants, which might have affected performance. Randomization produced two groups with similar characteristics in all measured attributes (age, experience, practice environment), suggesting that the arms were similar in this respect as well. Finally, 23% of the half of participants who provided feedback about the experience, complained about the game's verisimilitude. However, we found similar practice patterns on the game as compared with real-life, suggesting concerns about verisimilitude did not affect the external validity of the instrument. These complaints were also equally common in the two arms, hence should not have biased our results.

Improving the quality of physician performance is a high priority for multiple health care stakeholders. As a new source of insight into these processes, we offer a serious game that appears able to replicate the task environment, allowing us to manipulate cognitive load, and reveal the possible role of heuristic thinking on trauma triage decision making. Our results suggest that serious game technology has potential as a method of evaluating physician decision making and warrants further evaluation.

## Supporting Information

Appendix S1 Sample of feedback provided by participants after completing the game.
(DOCX)
