# Supplementary material for: Assessing the Validity of Using Serious Game Technology to Analyze Physician Decision Making
Source: PLoS One. 2014 Aug 25;9(8):e105445. doi: 10.1371/journal.pone.0105445 (PMC4143260; doi:10.1371/journal.pone.0105445)
Supplement: Appendix S1 — Sample of feedback provided by participants after completing the game. (DOCX) [file pone.0105445.s001.docx]

| Feedback | Examples |
| --- | --- |
| Positive | “This was an interesting program. The IT and administration interruptions were perfect!” (Participant 319) |
|  | “Enjoyable interactive module. Would be a great tool, and would be nice to have feedback or a list of cases that were part of the tool.” (Participant 232) |
|  | “Excellent. This was well done and enjoyable. Some slight stress and annoyance involved with the IT pager.” (Participant 180) |
| Negative - Verisimilitude | “Bit too long” (Participant 315) |
|  | “Rather unrealistic as we usually have a very clear idea of who we call, who we can admit, our available beds, and who we can transfer…It usually takes less than 1 minute to write admission orders for trauma or medicine. Here the ordering was very cumbersome and not exactly intuitive especially since we frequently order in sets (e.g. trauma panel, cardiac panel). (Participant 512) |
|  | “Only problem I had is that I felt I could not reassess any of the patients, ask them anything else I wanted to know.” (Participant 325) |
| Negative – Technical problems | “I had a lot of trouble seeing everything on the screen. For example a quarter of the text at the top of this screen is cut off by this text entry box.” (Participant 279) |
|  | “Interface occasionally froze using Internet Explorer. It was better with Chrome.” (Participant 414) |
|  | “I had a difficult time with the software at first. Clicking in an incorrect area (i.e. the black on the screen, the sound etc.) would freeze the system. Then I had to start over multiple times.” (Participant 344) |
